# Renal Sarcoidosis Presenting as Acute Kidney Injury With Persistent Hypercalcemia in the Absence of Respiratory Symptoms: A Report of a Rare Case

**DOI:** 10.7759/cureus.100591

**Published:** 2026-01-01

**Authors:** Muhammad J Khan, Muhammad Ali Akbar, Toqeer Ahmad, Aliza Zahid, Farhan Faisal

**Affiliations:** 1 Acute Internal Medicine, Midland Metropolitan University Hospital, Smethwick, GBR; 2 Acute Internal Medicine, HBS (Hazrat Bari Imam Sarkar) Medical and Dental College, Islamabad, PAK; 3 Acute Internal Medicine, Shifa International Hospital Islamabad, Islamabad, PAK

**Keywords:** acute kidney injury, denosumab, denosumab and sarcoidosis, extra pulmonary manifestations of sarcoidosis, renal sarcoidosis, sarcoidosis, sarcoidosis hypercalcemia, sarcoidosis treatment

## Abstract

Sarcoidosis is a chronic multisystem granulomatous disease of unknown etiology, known for its diverse manifestations and ability to mimic other pathologies. Renal involvement in sarcoidosis is uncommon.

We present a diagnostically challenging case of a 66-year-old male who presented with unintentional weight loss, anorexia, and lethargy, having acute kidney injury (AKI) and refractory hypercalcemia, ultimately diagnosed with renal sarcoidosis. This report underscores the significance of maintaining a broad differential diagnosis in cases of unexplained AKI and hypercalcemia, especially in the absence of respiratory symptoms.

Early recognition of key imaging and biochemical clues allows prompt initiation of therapy and helps optimize patient outcomes.

## Introduction

Sarcoidosis is a systemic granulomatous disease characterized by the formation of non-caseating granulomas in affected organs. Although pulmonary manifestations are most common, renal involvement, typically as granulomatous interstitial nephritis (GIN), is rare but clinically significant. Renal sarcoidosis can manifest with deranged calcium metabolism, nephrocalcinosis, or tubulointerstitial nephritis, sometimes progressing to renal failure [1]. Here, we diagnosed a rare case of renal sarcoidosis presenting with unintentional weight loss, anorexia, and lethargy, having acute kidney injury (AKI) and persistent hypercalcemia without significant respiratory symptoms.

## Case presentation

A 66-year-old man with a history of chronic obstructive pulmonary disease presented with unintentional weight loss, anorexia, and generalized fatigue. Vital signs and physical examination were unremarkable. Laboratory investigations revealed Stage 3 AKI with a serum creatinine of 299 µmol/L and an eGFR of 17 mL/min/1.73 m². Previous renal function was normal eight months prior. His regular medications include amitriptyline 10 mg at night, lansoprazole 30 mg twice daily, and two inhaled therapies: fluticasone furoate once daily and salmeterol 25 mcg, two puffs twice daily, which do not affect the serum corrected calcium levels.

Laboratory results are summarized in Table 1, which includes reference ranges and measurement units.

**Table 1:** Summary of Laboratory Findings Laboratory findings show AKI, hypercalcemia, and suppressed PTH levels. AKI: acute kidney injury, eGFR, estimated glomerular filtration rate; PTH, parathyroid hormone; ACE, angiotensin-converting enzyme.

Parameter	Result	Reference Range	Units
Serum creatinine	299	60–110	µmol/L
eGFR	17	>60	mL/min/1.73 m²
Serum corrected calcium	2.91	2.10–2.60	mmol/L
Phosphate	1	0.8–1.5	mmol/L
Parathyroid hormone (PTH)	<0.42	1.6–6.9	pmol/L
Vitamin D (25-OH)	16	50–125	nmol/L
ACE level	190	8–52	U/L

Imaging with renal ultrasound revealed bilaterally echogenic kidneys without obstruction. Despite IV fluid therapy, serum corrected calcium remained elevated (2.89-3.32 mmol/L), and renal function plateaued. Myeloma screening was negative.

**Figure 1:**
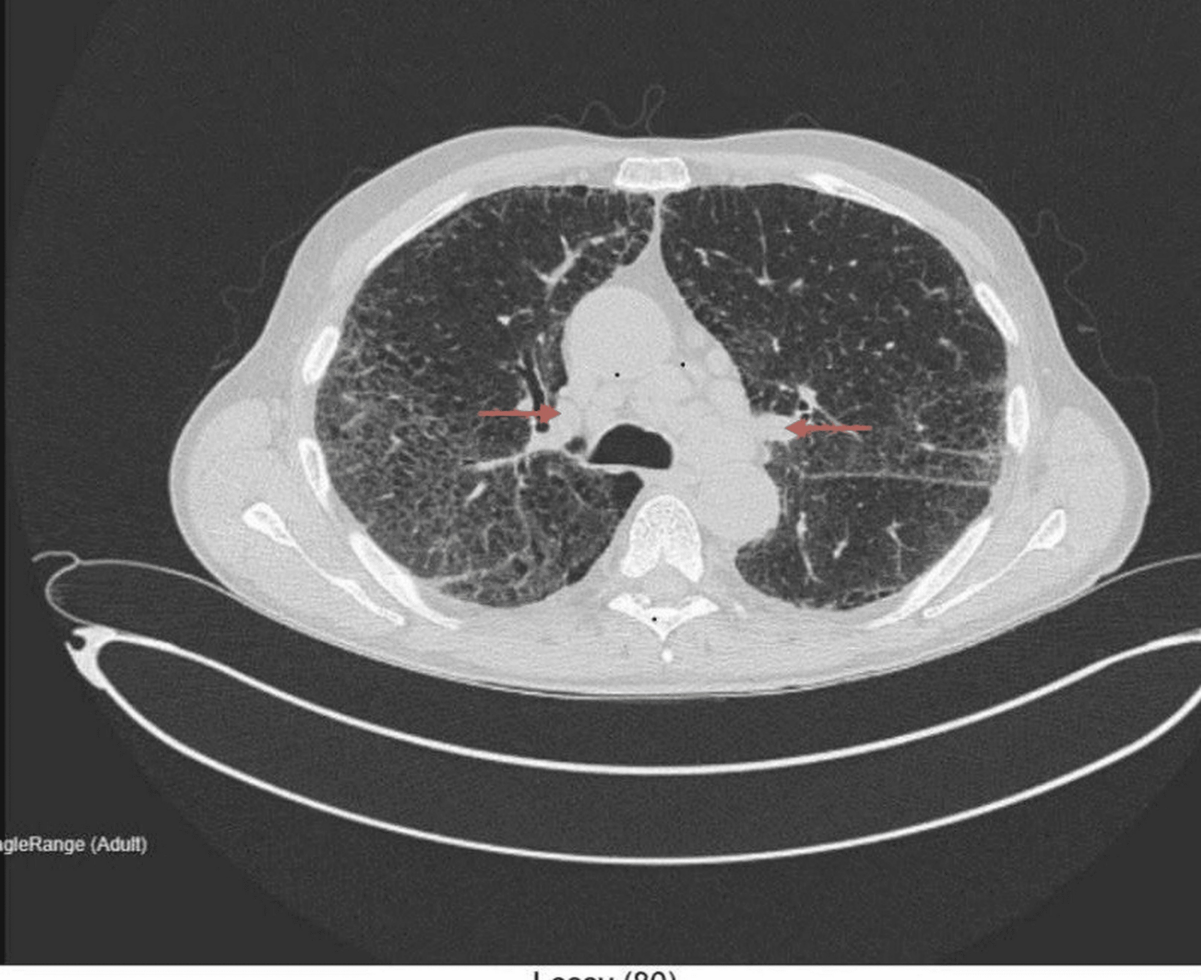
Axial non-contrast CT thorax, abdomen, and pelvis (lung window) Technique: Non-contrast computed tomography (CT). Findings: Widespread ground-glass changes with fibrotic interstitial lung alterations and multiple enlarged mediastinal and bilateral hilar lymph nodes are noted (red arrows). No pleural or pericardial effusion. Abdominal organs unremarkable, no lymphadenopathy or bone lesions. Conclusion: Fibrotic interstitial lung changes with indeterminate mediastinal hilar lymph nodes. No radiologic evidence of malignancy.

**Figure 2:**
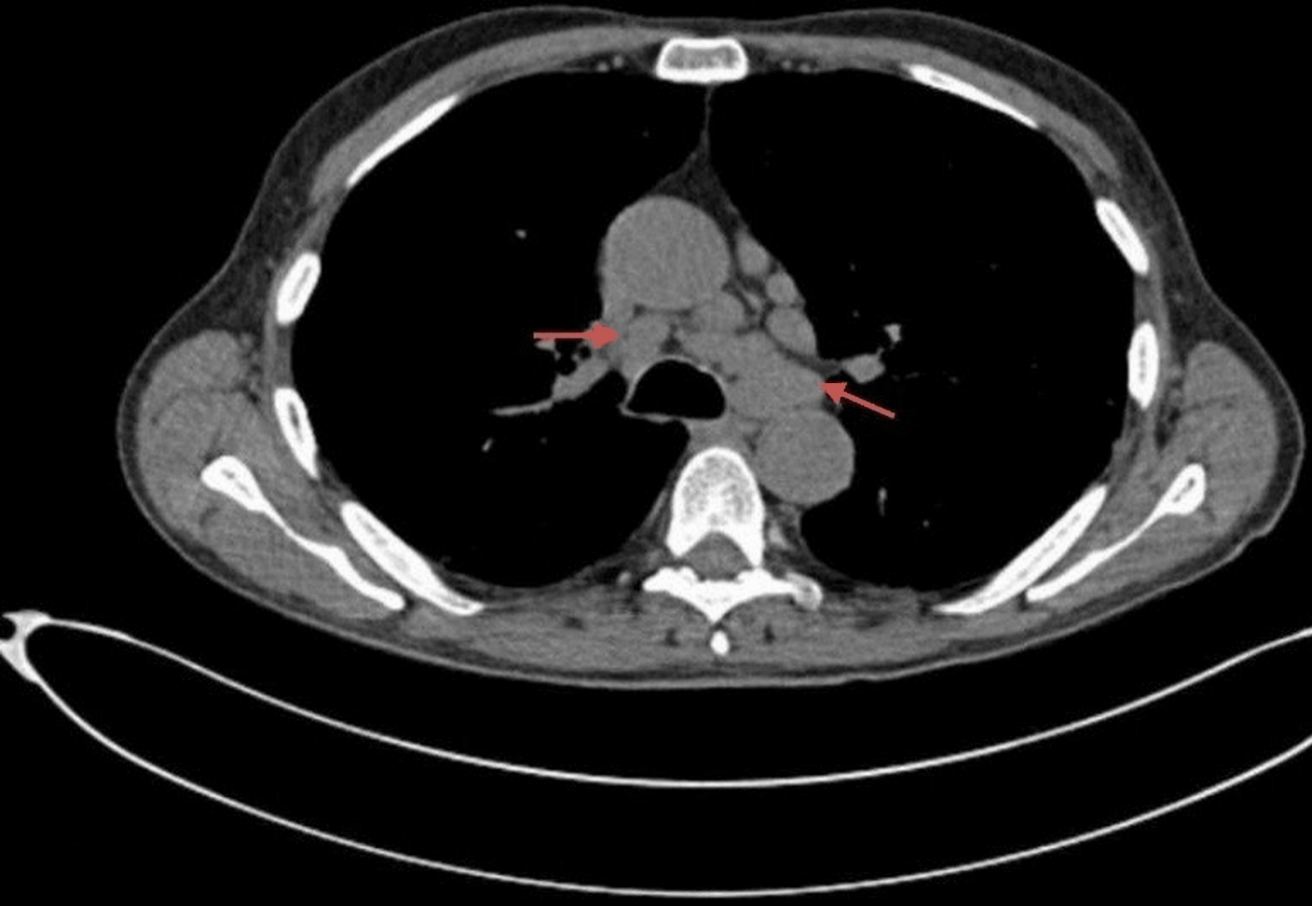
Non-contrast CT thorax. Technique: Non-contrast computed tomography (CT). Findings: Persistent fibrotic interstitial lung changes. Within the limitations of a non-enhanced study, no stigmata of malignancy were observed. Conclusion: Indeterminate mediastinal hilar lymph nodes.

A non-contrast computed tomography (CT) thorax, abdomen, and pelvis showed bilateral hilar lymphadenopathy, fibrotic interstitial lung changes, and ground-glass opacities with no evidence of malignancy (Figures 1, 2).

Elevated ACE levels raised suspicion for sarcoidosis. A renal biopsy confirmed granulomatous interstitial nephritis.

The patient was started on oral prednisolone 1 mg/kg/day and received subcutaneous denosumab (60 mg) after multidisciplinary consultation. Serum corrected calcium normalized to 2.59 mmol/L, and renal function improved (eGFR 35 mL/min/1.73 m²). Follow-up showed further renal recovery (eGFR 55 mL/min/1.73 m²) and stable calcium levels.

## Discussion

This report discusses a rare and complex case of granulomatous interstitial nephritis secondary to sarcoidosis, which presented primarily as unintentional weight loss, loss of appetite, and lethargy, having AKI and refractory hypercalcemia in a 66-year-old patient with a history of chronic obstructive pulmonary disease (COPD). The atypical presentation of this case and the difficulties in diagnosing sarcoidosis in the absence of explicit respiratory symptoms distinguish it from other cases.

The discussion will center on the clinical significance of this case, its diagnostic challenges, treatment strategy, and contribution to the existing literature.

The patient's presentation with unintentional weight loss, loss of appetite, and lethargy can be attributed to a variety of conditions. However, these symptoms did not immediately raise suspicion of sarcoidosis. Furthermore, the lack of significant findings on physical examination, combined with unremarkable respiratory symptoms, complicated the diagnostic process. The development of AKI with a markedly elevated serum creatinine level (299 µmol/L) and a decreased estimated glomerular filtration rate (eGFR) of 17 mL/min/1.73 m² was concerning. The patient's previous normal renal function tests made the acute deterioration more alarming, prompting an extensive workup. The finding of hypercalcemia with suppressed parathyroid hormone (PTH) levels was particularly significant, as hypercalcemia is a known complication of sarcoidosis, though it is not always the presenting feature.

The persistence of hypercalcemia despite aggressive intravenous fluid therapy indicated the need for further investigation into potential granulomatous diseases, such as sarcoidosis or malignancy. This case is unique because renal sarcoidosis as the primary manifestation is rare, and when it does occur, it frequently presents diagnostic challenges due to its nuances and the need to distinguish it from other causes of hypercalcemia and renal dysfunction, such as malignancy, which was initially suspected. The negative myeloma screening and subsequent CT scan, which revealed widespread ground-glass changes with fibrotic interstitial lung disease and mediastinal lymphadenopathy, suggested sarcoidosis, which was confirmed by elevated angiotensin-converting enzyme (ACE) levels and renal biopsy findings. Granulomatous interstitial nephritis (GIN) is a rare but well-known renal manifestation of sarcoidosis [2].

The pathogenesis includes the formation of non-caseating granulomas in the renal interstitium, which can cause varying degrees of renal impairment [2]. Sarcoidosis is thought to cause hypercalcemia because activated macrophages in granulomas produce more 1,25-dihydroxyvitamin D, which leads to increased intestinal calcium absorption and renal calcium reabsorption [3]. This mechanism explains why the patient's hypercalcemia persisted despite aggressive fluid management.

According to research, renal involvement in sarcoidosis, in any form (hypercalcemia, nephrolithiasis or nephrocalcinosis, tubular dysfunction, etc.), may occur in roughly 20-35% of patients. However, biopsy-proven granulomatous interstitial nephritis is much rarer and is reported in as few as 0.5-0.9% of all native kidney biopsies, though post-mortem series show GIN in about 7-23% of sarcoidosis cases, suggesting that GIN may be under-recognized during life [4].

Corticosteroids are typically used as the primary treatment for sarcoidosis-related renal involvement [5]. In this case, the patient was started on prednisolone at a dose of 1 mg/kg/day, which is the standard treatment for granulomatous interstitial nephritis [5].

Denosumab, a monoclonal antibody that inhibits osteoclast-mediated bone resorption, was used to treat refractory hypercalcemia and successfully normalized calcium levels [6].

This combination of therapies emphasizes the importance of a multidisciplinary approach in managing complex sarcoidosis cases that involve multiple systems. This case is particularly valuable to the literature because it demonstrates the importance of a comprehensive and systematic approach to diagnosis when dealing with unexplained AKI and hypercalcemia, especially in the absence of clear respiratory symptoms. It also emphasizes the significance of including sarcoidosis in the differential diagnosis of granulomatous diseases, even in the absence of typical presenting symptoms.

## Conclusions

This case illustrates a rare manifestation of sarcoidosis with predominant renal involvement, highlighting the diagnostic complexity in the absence of typical systemic features. Clinicians should maintain a high index of suspicion for sarcoidosis in patients presenting with unexplained AKI and hypercalcemia, even without pulmonary symptoms. Prompt diagnosis and treatment with corticosteroids and adjunctive therapies such as denosumab can lead to significant clinical improvement.
